# Supplementary material for: Fulvic Acid Improves Salinity Tolerance of Rice Seedlings: Evidence from Phenotypic Performance, Relevant Phenolic Acids, and Momilactones
Source: Plants (Basel). 2023 Jun 18;12(12):2359. doi: 10.3390/plants12122359 (PMC10301376; doi:10.3390/plants12122359)
Supplement: Supplementary file 1 [file plants-12-02359-s001.zip › plants-2411359-supplementary.pdf]

Article

Fulvic Acid Improves Salinity Tolerance of Rice Seedlings: Evidence from Phenotypic Performance, Relevant Phenolic Acids, and Momilactones

Supplementary Materials:

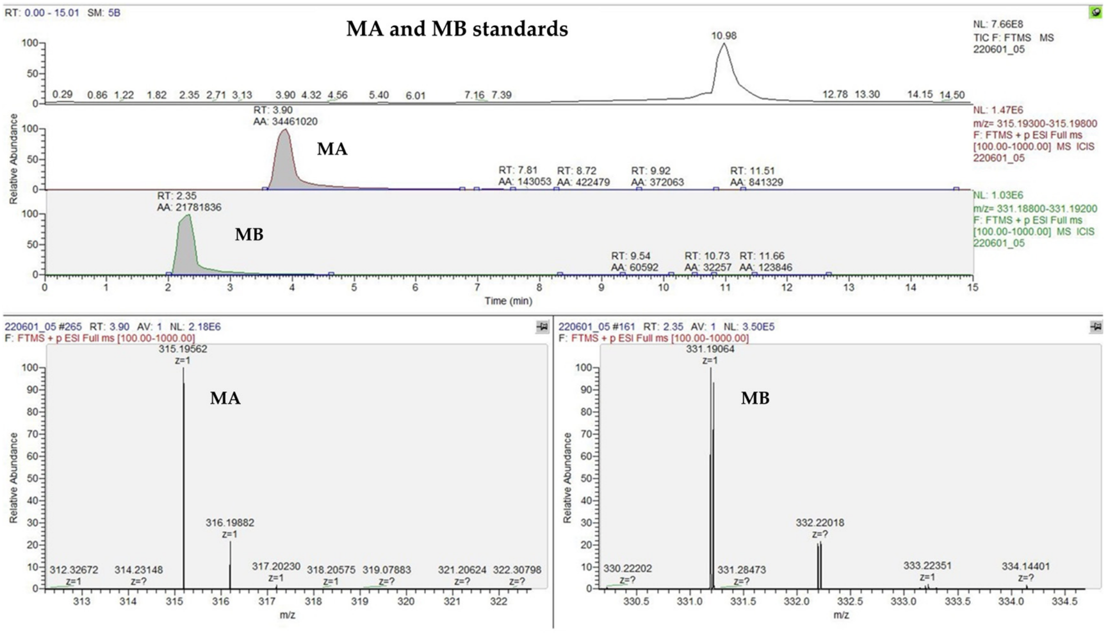

(a)

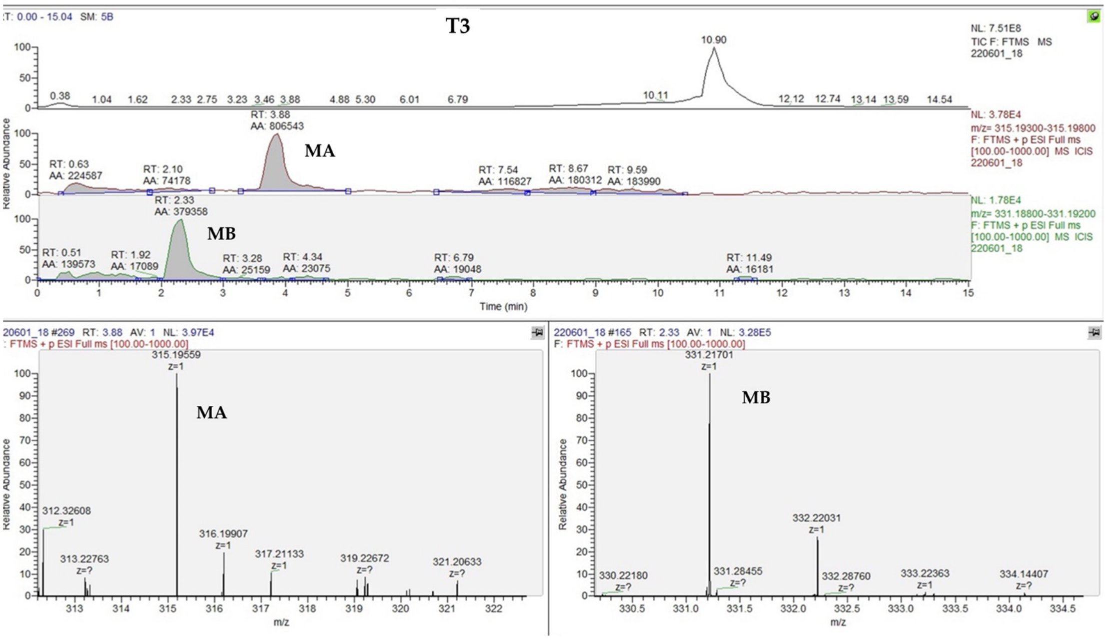

(b)

Figure S1. UPLC-ESI-MS chromatograms and mass spectra of MA and MB standards (**a**); and MA and MB detected in T3 treatment of Nipponbare seedlings (**b**).
